# The mass use of deltamethrin collars to control and prevent canine visceral leishmaniasis: A field effectiveness study in a highly endemic area

**DOI:** 10.1371/journal.pntd.0006496

**Published:** 2018-05-14

**Authors:** Bruna Martins Macedo Leite, Manuela da Silva Solcà, Liliane Celestino Sales Santos, Lívia Brito Coelho, Leila Denise Alves Ferreira Amorim, Lucas Edel Donato, Sandra Maria de Souza Passos, Adriana Oliveira de Almeida, Patrícia Sampaio Tavares Veras, Deborah Bittencourt Mothé Fraga

**Affiliations:** 1 LAIPHE – Laboratório de Interação Parasito-Hospedeiro e Epidemiologia, Instituto Gonçalo Moniz—FIOCRUZ, Salvador, Brazil; 2 Escola de Medicina Veterinária e Zootecnia, Universidade Federal da Bahia, Salvador, Brazil; 3 Instituto de Matemática e Estatística, Universidade Federal da Bahia, Salvador, Brazil; 4 Secretaria de Vigilância em Saúde, Ministério da Saúde, Brasília, Brazil; 5 Centro de Controle de Zoonoses de Camaçari, Secretaria de Saúde de Camaçari, Camaçari, Brazil; 6 Instituto de Ciência e Tecnologia de Doenças Tropicais (CNPq/MCT), Salvador, Bahia, Brazil; Universidade Federal de Minas Gerais, BRAZIL

## Abstract

**Background:**

Visceral leishmaniasis (VL) is a zoonosis of great importance. Limitations in current VL control measures compromise efficacy, indicating the need to implement new strategies. The aim of this study was to evaluate the effectiveness of the mass use of deltamethrin-impregnated collars in dogs as a public health measure to control and prevent canine visceral leishmaniasis (CVL).

**Methodology:**

An interventional study was implemented in two endemic areas in the district of Monte Gordo (Bahia-Brazil): an intervention area, in which VL seronegative dogs were collared, and a control area in which only conventional CVL control measures were applied. At baseline, seropositive dogs were removed and seronegative dogs were included. Dogs were then reevaluated every 7–8 months for almost two years. At each time point, dogs in the intervention area that remained seronegative received new collars and newly identified seronegative dogs were included and collared. The local zoonosis control authorities were notified of any dogs that tested seropositive in both areas, which were subsequently marked for euthanasia as mandated by the Brazilian Ministry of Health.

**Principal findings:**

In the first serological survey, seroprevalence was similar in both areas. At the second evaluation, significant reductions in seroprevalence were seen in both areas, while seroprevalence in the intervention area reduced to 6.0% during the final evaluation versus an increase of 11.0% in the control area. This significant increase and the estimated relative risk (RR = 0.55) indicated protection against CVL in the intervention area. Although CVL incidence did not differ significantly between the areas, an increased tendency was observed in the control area, which could be due to low seroconversion rates throughout the study or a high loss to follow-up.

**Conclusions/Significance:**

Although our evaluation of the effectiveness of deltamethrin-impregnated collars as a community-wide public health control measure was inconclusive, this measure likely provides protection over time. In endemic areas of Brazil, this strategy represents an operational challenge for local zoonosis control authorities, indicating the need for adjustments, including improved collar design.

## Author summary

Visceral leishmaniasis (VL) is a serious public health problem. Cases of canine VL (CVL) tend to precede cases of human VL and current control measures are ineffective in significantly reducing the number of disease cases. Therefore, in light of the need for new strategies, we evaluated the mass use of dog collars impregnated with deltamethrin as a public health measure to control and prevent CVL in a highly endemic region in Brazil. Although our results were inconclusive, they do point to the possible effectiveness of this measure if it were comprehensively implemented over an extended period of time. We identified the need to adjust the currently employed implementation strategy and call attention to the operational challenges faced by local zoonosis prevention authorities.

## Introduction

Visceral leishmaniasis (VL) is a chronic, systemic disease that represents a serious public health problem due to its wide geographic distribution and high lethality [1, 2, 3]. In the Americas, the VL zoonosis is caused by *Leishmania infantum* [4] and it is transmitted to humans and other mammals by the bite of sand flies [5]. *Lutzomyia longipalpis* is the main species of phlebotomine involved in VL transmission in Brazil [6, 7]. Because dogs are considered the main urban reservoir of *L*. *infantum*, they are crucially important in VL epidemiology, and therefore represent a prime target in VL control strategies [2, 8, 9, 10, 11].

The control measures currently employed by the Brazilian Ministry of Health for VL control consist of: I) early diagnosis and treatment of human cases; II) reduction of sand fly populations via the spraying of residual insecticides in domiciles; III) serological surveys of dogs and euthanasia of seropositive animals; IV) health education [12]. Studies showed that these control measures present significant limitations, which compromise its effectiveness and does not prevent the spread of the disease. Among these limitations, we can cite the high cost, opposition by dog owners, veterinary practitioners and animal protection institutions, a high turnover of dogs, and prolonged intervals between diagnosis and dog removal [2, 13]. Ashford *et al*. (1998) [14], Dietze *et al*. (1997) [15] and Souza *et al*. (2008) [16] have shown that these control measures are insufficient to control human and canine VL and emphasize the need to implement new intervention strategies that can more effectively contribute to VL prevention.

The testing of alternative control strategies including experimental studies has indicated that the use of collars impregnated with 4% deltamethrin represents a promising strategy for the individual protection of dogs against phlebotomine bites [17]. Killick-Kendrick *et al*. (1997) [18] and David *et al*. (2001) [19] reported that deltamethrin-impregnated collars protected dogs against an average of 96% of sand fly bites, which remained effective for eight months. Although the prevention of vector bites interrupts the *Leishmania* transmission cycle and prevents infection [20], few field studies have evaluated the efficacy of these collars in the context of control measures, or interference in the VL transmission cycle [21, 22, 23, 24]. Ribas *et al*. (2013) [25] and Sevá *et al*. (2016) [26] have used mathematical models to evaluate the effectiveness of disease control measures simulating the occurrence of VL in endemic areas. Both studies concluded that the use of insecticide-impregnated collars has a more significant impact on the prevalence of VL than euthanasia of seropositive dogs. These studies evaluated the use of deltamethrin collars and euthanasia separately as individual control measures; they did not evaluate these strategies jointly.

Field studies carried out in endemic areas in Italy [22, 23] and Iran [24] have shown that the use of deltamethrin-impregnated collars as a control measure for canine visceral leishmaniasis (CVL) significantly reduced the rate of seroconversion in canine populations. However, in these studied areas, *Leishmania* vectors were *Phlebotomus perniciosus* and *Phlebotomus kandelaki*, which differ from the principal vector in Brazil (*L*. *longipalpis*), and transmission of *Leishmania* does not occur year-round, due to seasonal dynamics affecting vectors, which is very dissimilar from what occurs in Brazil, where *L*. *infantum* transmission is continuous [27]. In Brazil, Reithinger *et al*. (2004) [28] conducted a field intervention study that lasted 5 months and used a mathematical model to estimate that, in places where the rate of transmission is high, the use of deltamethrin-impregnated collars would significantly reduce the risk of infection and it could be more effective than the strategy of euthanizing seropositive dogs. Camargo-Neves (2011) [21] showed that the use of these collars in seronegative dogs in association with the euthanasia of seropositive dogs resulted in a significant reduction in the incidence of CVL in an endemic municipality of São Paulo. However, these authors’ study design was limited with respect to the diagnostic technique employed, and they did not compare the results obtained from their intervention area with any controls.

Our study represents an initial attempt to evaluate, in real-world field conditions, the protective effect of the mass use of deltamethrin-impregnated collars to prevent CVL. We further compare the findings of this public health strategy, employed in combination with officially sanctioned prevention measures involving the euthanasia of seropositive dogs, to results from a control area in which deltamethrin collars were not used. The present study differs substantially from previously published reports, primarily due to the inclusion of a control area, as well as our focus on the canine population in general versus individual dogs and the effectiveness evaluation of collar use combined with the euthanasia of the seropositive animals. In addition, the present investigation was conducted in a highly endemic area for CVL, in conditions typically found in endemic areas throughout Brazil, i.e. year-round disease transmission, high canine population turnover, and large numbers of stray and semi-domiciled dogs. Therefore, this study effectively simulates the implementation of community-wide use of the deltamethrin-impregnated collar

Accordingly, the present study aimed to evaluate the effectiveness of community-wide implementation of 4% deltamethrin-impregnated collars in a highly endemic area of CVL together with official prevention measures, and compared these results with those obtained in a control area where only the euthanasia of seropositive dogs was performed.

## Methods

### Ethics statement

The present study was approved (Protocol n° 29/2013) by the Institutional Review Board for Animal Experimentation of the School of veterinary medicine of the Federal University of Bahia (UFBA). Serological testing results were forwarded to the Zoonosis Control Center (CCZ) in Camaçari (Bahia-Brazil), which is charged, with the collection of seropositive dogs to perform euthanasia as directed by the Brazilian Ministry of Health Decree n^o^ 51838. The dogs were euthanized according to the Brazilian Good Practice Guide for Euthanasia of Animals (Federal Council of Veterinary Medicine, 2012). All dog owners who agreed to participate in the study signed a term of free and informed consent.

### Study area

**Fig 1:**
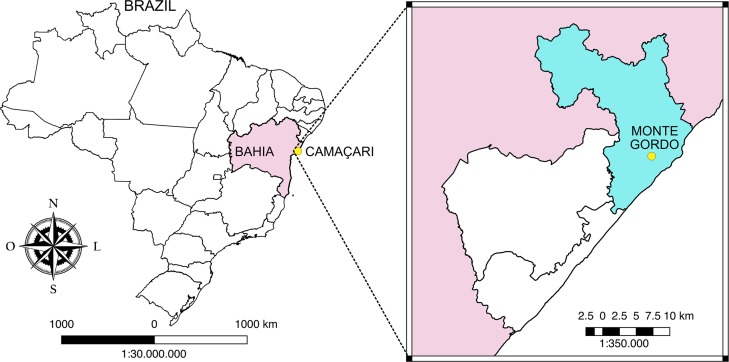
District of Monte Gordo in the municipality of Camaçari–Bahia–Brazil. Source: Terraview.

A longitudinal community trial study was performed between January 2014 and November 2015 in the district of Monte Gordo in the municipality of Camaçari (Bahia-Brazil) (Fig 1), an endemic area for VL in dogs and humans. Monte Gordo, located in the coastal region of Camaçari, has an estimated population of 29,573 inhabitants with 8,962 households [29]. From 2000 to 2008, a total of 69 human cases of VL were reported in the municipality of Monte Gordo, with an average of 8.6 cases per year according to the Health Department of the Municipality of Camaçari. In a cross-sectional study previously conducted in 2011–2012 using serological, parasitological and molecular CVL diagnosis evaluating a sample of 40 dogs, the prevalence of CVL was estimated at 57.5% in Monte Gordo (S1 Table).

### Study design and animals selection

**Fig 2:**
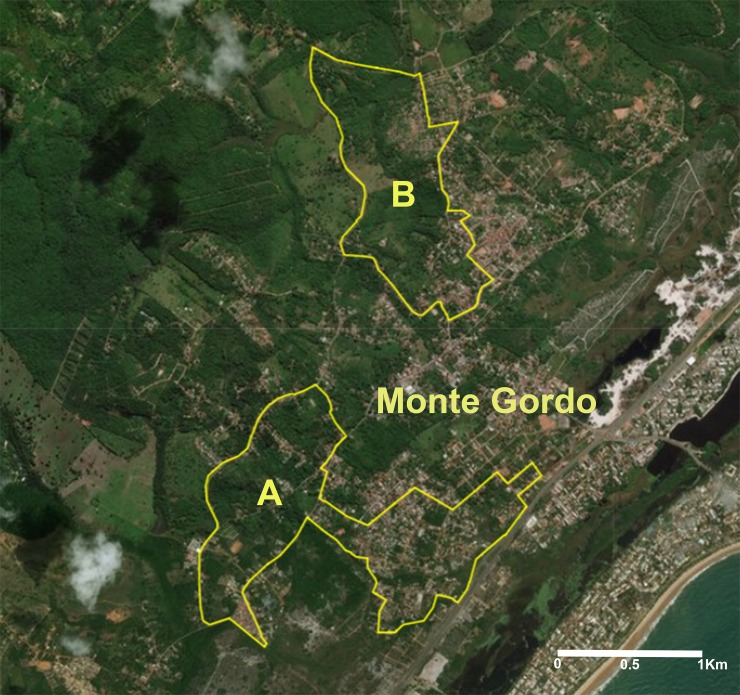
Identification of the intervention area (A) and control area (B) in the district of Monte Gordo–municipality of Camaçari (Bahia-Brazil). Source: USGS LandsatLook (https://landsatlook.usgs.gov/viewer.html).

Two areas were selected in the district of Monte Gordo: (A) an intervention area, in which VL seronegative dogs received a 4% deltamethrin-impregnated collar (Scalibor Protector Band, MSD) (collared dogs) and (B) a control area where VL seronegative dogs did not receive any collars (uncollared dogs) (Fig 2). Throughout the study, the veterinarians in charge of the leishmaniasis control program at the CCZ were notified of any dogs that tested seropositive in either area, which were subsequently marked for euthanasia as mandated by the Brazilian Ministry of Health.

The intervention and control areas were similar with regard to environmental characteristics. Although both areas were urban, native vegetation was present, in addition to livestock, including chickens and horses, around the perimeter of residences.

During the community trial study, three serological surveys were carried out, in each survey all residences in the two areas were visited and dog owners were informed of the study; if in agreement, they completed a form detailing animal and owner data used to identify the included dogs, which were then evaluated serologically.

Dogs residing in the intervention area that remained during all the study period were submitted to three serological evaluations approximately every 7–8 months, and those that were serologically negative received two 4% deltamethrin-impregnated collars replacements. Dog owners were instructed to notify the study staff of any signs suggestive of an allergic reaction to the collar (localized skin lesions, localized dermatitis or erythema, pruritus and hair loss). In these cases, a veterinarian was dispatched to visit the dogs and owners were advised to remove the collar if animal hypersensitivity was determined.

The collars that were lost were not replaced until the next serological evaluation, but the owners were advised of the importance of the animals keep the collars until the subsequent serological evaluation.

In the control area, VL seronegative dogs were reevaluated approximately every 7–8 months, but to these animals were not given collars. A total of three serological evaluations were performed on the animals that remained during the study period.

At the time of subsequent serological evaluations, all households in each area were revisited and any dogs present in the household that had not been included during the previous survey were then enrolled in the study (open cohort study design). Dogs that were considered lost to follow-up, i.e. not present during an evaluation, but were then encountered at an owner’s residence during the following survey, were then reevaluated and remained in the study. These dogs that were initially considered lost but returned to the study later are typically semi-domiciled dogs that have free access to the street and were not present in the house at the time of a serological evaluation but were present in the following survey.

The number of serological surveys and collar changes that each dog underwent varied due to the fact that the study is an open cohort, so not all the dogs included in the study remained from the beginning to the end, entering or leaving at different times.

### Sample collection and CVL diagnosis

All the dogs included in the present study, whether residing in the intervention area or the control area, were submitted to the current serological protocol mandated by the Brazilian Ministry of Health for CVL surveillance.

At the time of each serological evaluation, the animals were first screened using the DPP LVC (Bio-Manguinhos, Rio de Janeiro, Brazil) rapid test, administered in accordance with the manufacturer's recommendations at each dog owner's residence. Dogs from each study area that tested positive under DPP LVC were then submitted to venous blood collection for confirmation of CVL diagnosis by EIE LVC (Bio-Manguinhos, Rio de Janeiro, Brazil), in accordance with manufacturer recommendations. The CCZ authority was then notified of any confirmatory diagnostic results.

### Analysis

Databases containing animal identification and diagnostic results data were constructed using doForms 4.1.1 software (doForms Inc., USA), which were analyzed using EPI-INFO 7.2.0.1 (The Centers for Disease Control and Prevention—CDC, USA) and STATA 12 (StataCorp LP, USA) softwares.

The chi-square test was used to evaluate the homogeneity of the canine populations (collared and uncollared dogs) with respect to the following dog variables: sex, age group, breed and physical build (size) (Table 1).

**Table 1:** Canine characteristics recorded at baseline in each study area located in Monte Gordo–Camaçari (Bahia-Brazil).

Dog Characteristics	Intervention area, n/N (%)	Control area, n/N (%)	*p* value
**Sex**			
Male	225/404 (55.7%)	57/119 (47.9%)	0.163
Female	179/404 (44.3%)	62/119 (52.1%)	
**Age group**			
Puppy (0–1 year)	90/388 (23.2%)	22/116 (19%)	0.761
Young (1–2 years)	118/388 (30.4%)	42/116 (36.2%)	
Young adult (3–4 years)	82/388 (21.1%)	26/116 (22.4%)	
Adult (4–7 years)	62/388 (16.0%)	19/116 (16.4%)	
Old (> 7 years)	36/388 (9.3%)	7/116 (6.0%)	
**Breed**			
Mixed-breed	306/386 (79.3%)	98/115 (85.2%)	0.200
Purebred	80/386 (20.7%)	17/115 (14.8%)	
**Size**			
Small (0.1–10 kg)	185/398 (43.5%)	53/116 (43.1%)	0.933
Medium (11–20 kg)	171/398 (43.0%)	53/116 (45.7%)	
Large (21–40 kg)	42/398 (10.3%)	10/116 (8.6%)	

**Fig 3:**
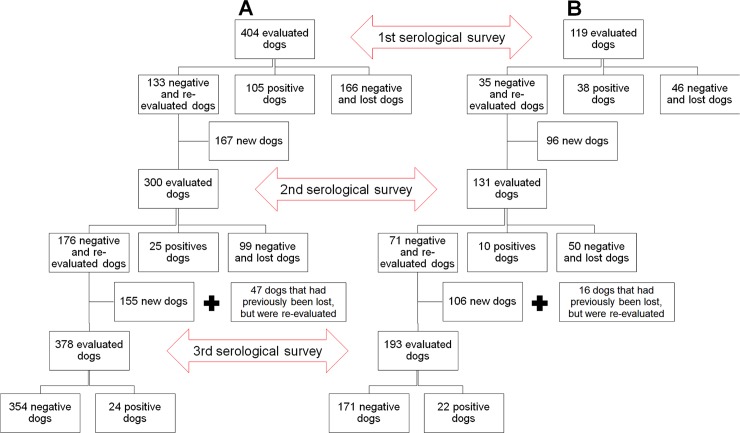
Number of dogs, and serological and inclusion status of the open cohort in intervention (A) and control (B) areas.

CVL seroprevalence was calculated for a given study area at the time of each serological survey. Our seroprevalence analysis considered canine populations of both areas as an open cohort, i.e. dogs that were lost to follow-up and those that returned to the study, as well as new dogs, were included in each calculation (Fig 3).

Each study area’s CVL incidence rate was calculated following the second and third surveys, but for this analysis canine populations of both areas were treated as closed cohorts, i.e. only dogs identified with negative serology at the time of the first survey were then followed in the subsequent evaluation periods throughout the study.

Comparisons between seroprevalence and incidence rates among the intervention and control areas were performed using the chi-square test (Χ^2^). The effectiveness of the mass use of deltamethrin collars was estimated by calculating relative risk (RR) for CVL in collared versus uncollared dogs. The protective effect of the mass use of the deltamethrin collar (effectiveness) was then evaluated by calculation of: 1 –RR.

Cumulative incidence was calculated as proposed by Morgenstern et al. (1980) [30]. For this evaluation we have to consider the time in which each animal participated in the study, since we did not have this data, we estimated the time, using half of the time between surveys for animals entering or leaving during the study, and the complete period of time when the dogs remained for the entire period between surveys in the study.

Survival analysis was used to compare the distribution between study areas (intervention vs. control areas) with respect to the periods between a given animal’s initial seronegative diagnosis and the occurrence of seroconversion. Survival analysis considered only animals that were present at two or more subsequent evaluation points, i.e. those with negative CVL serology at an initial time of survey (e.g. first or second), as well as newly seronegative animals included in subsequent serological surveys. The event of interest was seroconversion. Censoring occurred due to the death of an animal or when they were absent at a subsequent survey interval. Kaplan-Meier's nonparametric method was used to estimate survival function, depicted as survival curves, for each study area. Wilcoxon’s test was used to evaluate differences in survival between the areas. For all analyses, results were considered statistically significant when p ≤ 0.05.

## Results

The first serological survey (baseline) occurred between January 2014 and April 2014 in the intervention area and between May 2014 and July 2014 in the control area. A total of 404 domiciled and semi-domiciled dogs were initially enrolled in the intervention area versus 119 dogs in the control area. The second and third serological surveys took place in the intervention area between August/2014 to March/2015 and April/2015 to September/2015, respectively, versus between March/2015 to May/2015 and September/2015 to November/2015 in the control area.

Descriptive data regarding the animals evaluated at baseline are shown in Table 1. No significant differences were seen between the characteristics of each canine population at baseline, nor in later survey evaluation periods (Table 1).

Fig 3 summarizes the number of animals, in addition to serological and inclusion status, evaluated throughout the community trial study in both the intervention area (A) and the control area (B), each considered as an open cohort.

**Fig 4:**
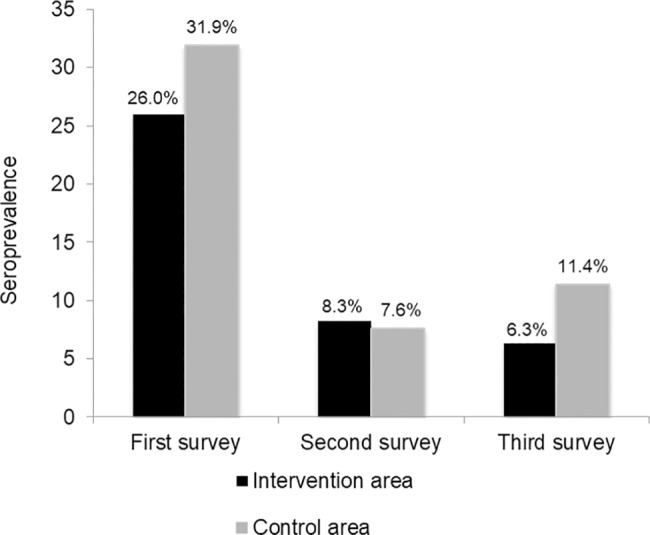
Seroprevalence of CVL in study intervention and control areas.

At the time of the first serological evaluation, 65.6% (265/404) of the dogs in the intervention area tested negative for CVL under DPP LVC and received the collar. Among dogs that were positive by DPP LVC, 26.0% (105/404) were confirmed by EIE LVC and the CCZ was duly notified. Dogs that showed negative results under EIE LVC were then revisited and received a collar. In sum, 299 dogs in the intervention area received a deltamethrin-impregnated collar upon completion of the first survey. In the control area, 61.3% (73/119) presented negative results under DPP LVC at the time of the first survey, while 38.7% (46/119) were positive. A total of 31.9% (38/119) of these animals were then confirmed by EIE LVC, resulting in CCZ notification. Again, dogs with negative confirmation by EIE LVC continued to be monitored at subsequent survey periods. Accordingly, the initial CVL prevalence was estimated at 26.0% (105/404) in the intervention area and 31.9% (38/119) in the control area (Fig 4).

During the second survey, 167 new dogs were serologically evaluated in the intervention area, while only 44.5% (133/299) of the negative animals in the first survey were reevaluated, due to substantial loss to follow-up (Fig 3 and S2 Table). In the control area, 96 new dogs were evaluated, and testing was repeated in 43.2% (35/81) of the animals with previously negative results (Fig 3). Thus, the second survey included a total of 300 animals in the intervention area and 131 in the control area (Table 2).

**Table 2:** Number of dogs evaluated in each study area during three survey periods.

Area	Evaluated dogs	Number of dogs, n/N (%)		
		1^st^ survey (*Baseline*)	2^nd^ survey	3^rd^ survey
Intervention	Reevaluated	---	133/300 (44.3)	223/378 (59.0)
	Newly included	404	167/300 (55.7)	155/378 (41.0)
Control	Reevaluated	---	35/131 (26.7)	87/193 (45.1)
	Newly included	119	96/131 (73.3)	106/193 (54.9)

In the second survey, serological evaluation revealed 82.3% (247/300) of the dogs in the intervention area as negative, and all these animals received a new collar. Dogs that tested positive, 17.7% (53/300), were submitted to confirmatory testing under EIE LVC, and CVL was confirmed in 8.3% (25/300); accordingly, 28 dogs of these 53 animals were then collared (Fig 3). Altogether, 275 dogs in the intervention area received new deltamethrin-impregnated collars during the second survey. In the control area, 88.5% (116/131) of the dogs were negative, versus 11.5% (15/131) with positive screening results, 7.6% (10/131) of which were confirmed by EIE LVC (Fig 3). Accordingly, a total of 121 negative dogs in the control area continued to be monitored at the subsequent time of evaluation.

In the third and final serological survey, a total of 378 dogs were evaluated in the intervention area, 155 of which were newly included (Table 2). In all, 84.7% (320/378) of these animals were negative and thusly received a new collar, while CVL was confirmed in 6.3% (24/378) by EIE LVC, resulting in 34 additional dogs being collared. Accordingly, a total of 354 dogs in the intervention area received a new deltamethrin-impregnated collar at the time of the third survey. In the control area, a total of 193 dogs were evaluated, 106 of which were new (Table 2), and 80.8% (156/193) of these dogs tested negative. With respect to CVL-positive dogs, 11.4% (22/193) were confirmed by ELISA.

In all, the study evaluated a total of 726 dogs in the intervention area and 321 in the control area. The average number of dogs lost to follow-up throughout the study was high in both areas (30.0% in the intervention area and 25.0% in the control area).

Throughout the community trial study, a total of 928 deltamethrin-impregnated collars were placed on dogs with negative serology: 299 dogs in the first survey, 275 in the second survey and 354 in the third survey.

Due to the fact that most of the animals are semi-domiciled and have free access to the street, collar losses during the study were high, up to 64.7% among the dogs reevaluated during subsequent survey periods. Less than 42.0% of the animals with repeated serological results retained their collars at the subsequent evaluation (Table 3).

**Table 3:** Number of dogs in the intervention area that remained collared until the subsequent survey.

Time of evaluation		n/N (%)
1^st^ survey (*Baseline*)	Dogs receiving collar	299
2^nd^ survey	Dogs reevaluated	133/299 (44.5%)
	Dogs that remained collared	47/133 (35.3%)
	Dogs receiving new collar	275
3^rd^ survey	Dogs reevaluated	176/275 (64.0%)
	Dogs that remained collared	73/176 (41.5%)
	Dogs receiving new collar	354

In the first survey, one animal was observed to present an allergic reaction to the collar, and the owner removed the animal from the study. In the second survey, two dogs from this same owner also showed signs of allergic reaction and were withdrawn. In the third survey, seven dogs that had been previously evaluated had their collars removed because of reported signs of allergic reaction, but these owners agreed to the reevaluation of these animals without the placement of new collars.

Although the seroprevalence of CVL in the intervention area was numerically higher than in the control area at the time of the first survey, no statistical difference was detected between the two study areas (Table 4). In the second serological survey, a significant reduction in seroprevalence was seen in each area in comparison to the first survey, despite no statistical difference in seroprevalence between the study areas. At the time of the third survey, a significant reduction in seroprevalence was observed in the intervention area compared to both prior surveys, as well as the seroprevalence detected in the control area in the third survey. In addition, the seroprevalence in the control area during the third survey was found to be significantly higher than in the second. Accordingly, the calculated relative risk (RR) suggested that compared to uncollared dogs, collared animals had 44.7% of effectiveness against CVL at the time of the third survey (Table 4).

**Table 4:** Estimated effect of deltamethrin collar intervention on CVL seroprevalence at the time of each serological evaluation.

Evaluation time		Intervention area	Control area	RR (95% CI)	Effectiveness (%)
1^st^ survey (*Baseline*)	Evaluated dogs	404	119	0.81 (0.60–1.11)	
	Positive dogs	105	38		
	Seroprevalence (%)	26.0^(a)^	31.9^(b)^		
2^nd^ survey	Evaluated dogs	300	131	1.09 (0.54–2.21)	
	Positive dogs	25	10		
	Seroprevalence (%)	8.3^(a)^ ^(c)^	7.6^(b)^ ^(d)^		
3^rd^ survey	Evaluated dogs	378	193	0.56 (0.32–0.97)	
	Positive dogs	24	22		44.7
	Seroprevalence (%)	6.3^(c)^	11.4^(d)^		

RR = relative risk and CI = confidence interval

^(a)^ RR = 3.12 (CI = 2.07–4.70)

^(b)^ RR = 4.18 (CI = 2.18–8.02)

^(c)^ RR = 1.31 (CI = 0.77–2.25)

^(d)^ RR = 0.67 (CI = 0.33–1.37)

^(a)^ and ^(b)^ comparative analysis between the first and the second surveys within the same study area

^(c)^ and ^(d)^ comparative analysis between the second and the third surveys within the same study area

**Fig 5:**
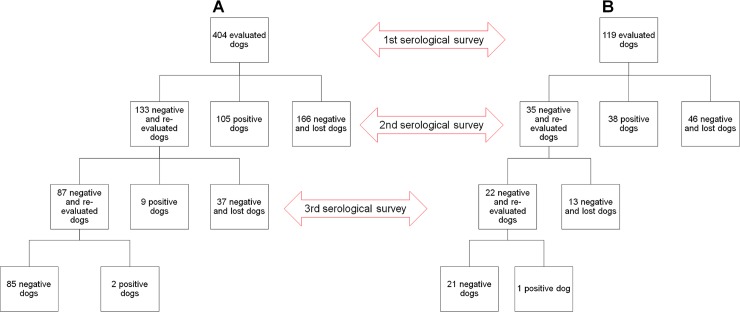
Number of dogs, and serological and inclusion status of the closed cohort in intervention (A) and control (B) areas.

The closed cohort was used to determine and compare CVL incidence among the study areas throughout the survey times, i.e. dogs that were newly included in the second and third surveys, as well as dogs not evaluated in the second survey, were considered lost to follow-up and thusly were not included in this analysis (Fig 5). Accordingly, among the 404 dogs initially evaluated in the intervention area and the 119 dogs in the control area, a total of 299 and 81 VL seronegative dogs were respectively included in the closed cohort.

At the time of the second survey in the intervention area, serological evaluations were repeated in 44.5% (133/299) of the dogs originally included. Of these, 93.2% (124/133) tested negative and received a new collar, while 6.8% (9/133) were positive, resulting in CCZ notification (Table 5). In the third survey, serological evaluations were repeated in 87 dogs, resulting in 97.7% (85/87) seronegativity versus 2.3% (2/87) with positive serology for CVL (Table 5). At the time of the second survey in the control area, serological evaluations were repeated in 43.2% (35/81) of the dogs originally included, yet none had positive serology. In the third survey, 22 dogs were reevaluated, with 95.5% (21/22) testing seronegative and 4.5% (1/22) presenting seropositivity for VL (Table 5).

**Table 5:** Estimated effect of deltamethrin collar intervention on CVL incidence.

Evaluation time		Intervention area	Control area	RR (95% CI) ^(1)^	Effectiveness (%)
2^nd^ survey	Positive dogs	9^(2)^/133	0/35		
	Incidence (%)	6.8	-		
3^rd^ survey	Positive dogs	2/87	1/22	0.51 (0.05–5.33)	49.0
	Incidence (%)	2.3	4.5		

^(1)^ RR = relative risk and CI = confidence interval

^(2)^Two of these animals presented inconclusive serology in the first survey, i.e. each was positive under DPP at baseline, but without EIE LVC confirmation

CVL incidence in the closed canine cohort reduced in the intervention area between the second and third surveys, while incidence increased in the control area. The protective effect (effectiveness) of the deltamethrin collar was estimated at 49.0% (Table 5), yet this was not statistically significant. Further evaluation of CVL incidence in the intervention area stratified according to the animals that remained collared throughout both subsequent serological surveys revealed no statistical differences.

Considering the closed canine cohort and estimating the period of time dogs were at risk to be infected, while in the study, the cumulative incidence showed similar results between intervention and control areas (0.0002 positive dogs/day).

**Fig 6:**
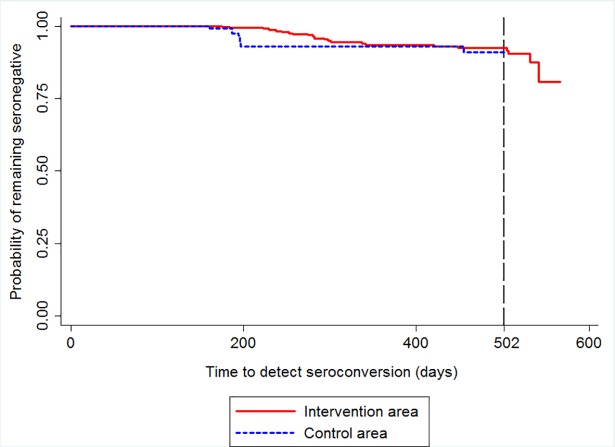
Survival curve indicative of elapsed time from initial seronegative diagnosis to seroconversion, stratified by study area.

In the survival analysis we considered only animals that were present at two or more evaluation points. In this way, 299 dogs that tested negative in the first survey were considered in the intervention area, plus the 151 new dogs that were included in the second survey and were negative at their inclusion, totalling 450 dogs for the analysis using the survival curve. Animals that had already started with a positive result in the first survey and the new ones that were included in the second survey and had a positive result at the time of inclusion were not considered for survival curve analysis. Following the same selection criteria, in the control area, 81 dogs were considered in the first survey and 86 dogs in the second survey, totalling 167 dogs. Of the 450 dogs in the intervention area, nine seroconverted in the second survey and 15 in the third survey. Among the 167 dogs in the control area, 11 were seroconverted in the third survey. Thus, a total of 24 dogs seroconverted in the intervention area versus 11 in the control area. Fig 6 depicts survival curves illustrating the probability of remaining seronegative up to time t for dogs in each study area. Kaplan-Meier analysis indicated that 4.2% of the animals were at risk of CVL seroconversion in the intervention area, versus 7.0% in the control area after 284 days of follow-up, interval that represents half of the study time considered for the survival analysis. After 502 days of follow-up, when the study was completed in the control area, there were 101 seronegative dogs in the intervention area. At that time, the risk of seroconversion was 7.6% in the intervention area and 8.9% in the control area. The survival curves demonstrated a longer survival time among dogs in the intervention area. The Wilcoxon test indicates difference in the survival function between study areas (χ2 = 5.78; p = 0.02).

## Discussion

VL continues to be a major public health concern throughout the world, despite the extensive amount of research that has been conducted over the years [31]. As a result, novel alternatives for VL control are being tested. The Brazilian Ministry of Health has instructed different research groups to carry out field studies in several Brazilian states endemic for VL in order to evaluate the effectiveness of the use of deltamethrin collars as a community-wide control measure to reduce the prevalence and incidence of CVL.

In the present study areas, the seroprevalence of CVL evaluated at baseline was similar to seroprevalence of 21.7% reported by a cross-sectional study conducted by Julião *et al*. (2007) [32] in a canine population from the same endemic area. In the second survey, seroprevalence decreased significantly in both study areas, possibly due to the culling of approximately 140 seropositive dogs following CCZ notification. This marked decrease in prevalence was attributed to the euthanasia of the animals since, in the normal routine of control actions of the municipality, euthanasia is not performed promptly after diagnosis and in a high number of dogs in a short period of time. Changes implemented in this routine at the study period resulted in faster euthanasia, different from the normal routine what we believe has impacted the reduction of the disease in both areas. In the third survey, after the impact of the removal of positive dogs, the seroprevalence in the intervention area was significantly lower in comparison to the control area.

We believe that the increase of the seroprevalence in the third survey has been the result of collar effect because we evaluated two possible bias: i) different number of new animals in each area during the study (96 dogs included in the control area and 167 in the intervention area). Although, these total numbers were different, we do not detected statistical differences in the percentage of positive dogs: in the control area we found 10.4% (10) positive dogs vs. 89.6% (86) negative and in the intervention area, 9.6% (16) tested positive vs. 90.4% (151) that tested negative, and ii) animals which were previously loose and reevaluated and may be exposed by the disease agent during the whole period of the study. Evaluating the dogs considered as lost but returned to the study between the second and third surveys, we observed that out of the 16 dogs in this situation in the control area no one seroconverted, while out of the 47 of the intervention area, four seroconverted. In this way, our data indicate that the observed seroprevalence increase in the control area did not occur due to the inclusion of the new dogs, re-inclusion of animals, or because they were positive. In fact, we believe this result reinforces the possibility of the difference between the prevalences of intervention and control areas have been the result of collar effect.

A longitudinal study, conducted by Camargo-Neves (2011) [21] in an endemic area in São Paulo, Brazil, also evaluated the effectiveness of the deltamethrin collar in conjunction with official local control measures, and reported a significant reduction from in CVL seroprevalence, from 10.9% to 4.2%. However, it is important to note that these authors did not employ a control group for comparison purposes.

Our results indicate that the incidence of CVL tended to reduce in the intervention area, in contrast to the increasing trend observed in the control area. We estimated that the effectiveness of the deltamethrin collar offered a protection rate of around 45.0%. These findings are similar to those reported by Maroli *et al*. (2001) [33] in an evaluation conducted in Italy after an initial transmission season, in which a CVL protection rate of 50.0% among dogs was estimated in an intervention area compared to control areas. However, due to seasonal VL transmission in these authors’ region, they employed an evaluation period lasting for two consecutive transmission seasons. Similarly to our results, differences in CVL incidence between the areas were not statistically significant in the first transmission season. After the second transmission season, these authors reported an increase to 86.0% in CVL protection among the collared dogs, with statistical significance. Accordingly, this leads us to speculate that our study period was insufficient to accurately assess the effectiveness of the deltamethrin collar in reducing the incidence of CVL. Although the transmission in Brazil occurs all year round, there is a seasonal variation of the VL vector population that affects the transmission rate; it may reduce transmission but not stop it [34, 35]. Taken together, these findings offer persuasive evidence that protection against CVL in endemic areas will likely rise over time as a consequence of subsequent collar replacement in addition to the application of new collars in dog populations, resulting in a reduction in the proportion of infected dogs. Furthermore, seronegative collared dogs will gradually replace seropositive ones, essentially reducing the susceptible canine population. Another study in Italy found an 84.0% decrease in CVL incidence after two transmission seasons among kenneled deltamethrin-collared dogs [22]. In Iran, Gavgani *et al*. (2002) [24] also found a significant reduction in CVL incidence among dogs using deltamethrin collar.

The high loss of follow-up (approximately 50.0%) that occurred in the present study, in most cases was caused by the death, move, or disappearance of the animal, together with a relatively low number of dogs that seroconverted in the second (n = 9) and third surveys (n = 3) (Fig 5). This may have influenced our CVL incidence results as well as the survival analysis. Moreover, we also encountered several other difficulties that likely had a negative influence on our evaluation of the effectiveness of using deltamethrin collars as a mass public health measure:

i) A delay of 2–3 months in the scheduled reevaluation of included dogs, at which time collars were replaced in the intervention area. At the outset, an ideal interval of six months was established for collar replacement, reflecting the time of effectiveness of these collars; yet several issues (worker strikes, lack of regular funding, reduction in staff availability, etc.) occurred throughout our study that directly affected the operations of health CCZ agents who were essential to carrying out fieldwork. These challenges triggered delays in the second and third serological surveys, resulting in an average evaluation interval of eight months. This delay may have compromised the effectiveness of this control measure, as David *et al*. (2001) [19] showed that the repellent effect against *Lu*. *longipalpis* of these collars remains above 90.0% after eight months of use; however, its lethal effect reduced to 35.0%, gradually declining after six months of use.
ii) A high number of previously unevaluated dogs regularly appeared in the intervention area in subsequent surveys (see Fig 5), and these animals were unprotected until the time of their subsequent inclusion. Delays in the evaluation of these new dogs likely affected the effectiveness of this control measure, as previously reported by Gavgani *et al*. (2002) [24] and Reithinger *et al*. (2004) [28]. These authors showed that the efficacy of deltamethrin-impregnated collars as a VL control measure is highly dependent on coverage rate, necessitating the rapid collaring of new dogs introduced into the canine population.
iii) We found a high number of dogs in our intervention area that lost their collars, consequently remaining unprotected until the subsequent evaluation. The dogs residing in Monte Gordo are generally mixed-breed, semi-domiciled animals, and typically are allowed to roam free in the area surrounding the owner’s residence, which facilitated the loss of collars between dogs in the intervention area and loss of follow-up in the two study areas. This high rate of collar loss (up to 64.7%) certainly impaired effectiveness. According to Reithinger *et al*. (2004) [28], the impact of using deltamethrin collars as a CVL control measure is dependent on their collective use and loss rate. Additionally, Gavgani *et al*. (2002) [24] reported that the collar replacement and the maintenance of a high coverage rate throughout the VL transmission period is determinant in the maintenance of control measure effectiveness. Both of these authors commented on this potential disadvantage in regions where VL transmission occurs throughout the year, as is the case in Brazil. In this study, we did not replace the lost collars before the next survey, since we decided to simulate the real conditions of application of the collar as a community public health measure. This was the design of the study requested by the Ministry of Health, considering that if this measure control of VL would be applied by the government in a certain community, it will not be possible to perform replacements of each lost collar before the expected period of 06 months. This impossibility is due to logistic difficulties, high costs and also for being a region where the population has low income, in addition to the high price of the collar that can be sold by the owner.
iv) The delay in the euthanasia of seropositive dogs, resulting in the maintenance of these animals in the study areas. The CCZ authorities were immediately notified with respect to all dogs with positive serology. According to Brazilian Ministry of Health protocols, agents are instructed to collect and euthanize these animals in a timely manner. However, throughout our study, the operational problems faced by the CCZ resulted in the delay of the removal of seropositive animals from the study areas. Although it was not possible to obtain records from the CCZ regarding the number of dogs that were collected, nor an accurate assessment of the interval between diagnosis and euthanasia, we observed in our final survey that no less than 15 out of 130 dogs with positive serology from the previous surveys remained in the intervention area, and 18 out of 48 were reevaluated in the control area. Obviously, these animals represent sources of infection for the phlebotomine vector and play a determinant role in the eventual spread of disease, thereby compromising the effectiveness of this control measure. The delay in the euthanasia of seropositive dogs was a complication because we were evaluating the combination of deltamethrin collar with the euthanasia of seropositive dogs and this bias may impact in the effectiveness evaluation.

Variance in phlebotomine density among the study areas may also have influenced our evaluation of collar effectiveness. A concomitant study conducted by our research group evaluated phlebotomine density among the two study areas and found that, between April and July 2015, monthly captures of sand flies demonstrated 30 *Lu*. *longipalpis* retrieved from the intervention area versus 11 *Lu*. *longipalpis* from the control area (S3 Table). This suggests a higher VL transmission power in the intervention area where more sandflies were captured, which has influenced the results observed herein.

Another limitation of our study was the sample size. We calculated a sample size with 80% power; unfortunately, however, as the study was unfolding in the field, there were follow-up losses that lowered the power of the study to approximately 60%, once we had difficulties in conducting the study, mainly due to problems with the human resources and the structure of the public health system employed as mentioned above.

In light of the difficulties mentioned above, which either could or should have significantly affected our evaluation of the effectiveness of the community-wide use of deltamethrin-impregnated collars a control measure in an area endemic for CVL, we can conclude that the implementation of this control measure in countries such as Brazil proves challenging. This is not only because disease transmission occurs year-round, but also due to a high turnover in the canine population, in addition to elevated numbers of stray and semi-domiciled dogs. Accordingly, we must emphasize that the success of this community-wide strategy depends not only on the efficacy of the collar itself, but also on the adoption of other control measures in parallel, in addition to other variables, especially considering that the majority of the canine population in endemic area is semi-domiciled. Furthermore, we recommend improvements in collar design to reduce the substantial loss rates seen herein, as high collar coverage rates must be maintained in order to have a significant effect on *Leishmania* transmission rates. This entails not only the rapid replacement of lost collars, but also rapid collaring of newly recruited dogs into the canine population.

Effective control of CVL depends on many factors: the maintenance of high rates of protective collar use, the rapid withdrawal of seropositive dogs from the canine population in endemic areas, and intensive public health education to raise awareness of the importance of responsible ownership and collar maintenance. Thusly, we can conclude that in countries faced with endemic disease and large stray and semi-domiciled dog populations, the strategy of community-wide use of deltamethrin collars as applied in the present study may not be thorough enough.

In sum, the use of 4% deltamethrin impregnated collars as a mass control measure did not present conclusive results, although many findings, such as significant differences in CVL prevalence, a protection rate of 45.0% against CVL and a tendency towards a reduction in CVL incidence in the intervention area are all indicative of increased protection in the collared dog population studied.

Accordingly, we recommend further community trial studies involving several important modifications to the present protocol: 1) an altered study design (longer evaluation period, more regular survey intervals, and improved promptness in the removal of dogs); 2) improvements to the collar (greater durability and better buckle design); 3) adaptive measures to overcome shortcomings of the municipal zoonosis control authority.

## Supporting information

S1 Table Prevalence found in a cross-sectional study previously conducted between the years 2011 and 2012 in Monte Gordo. (DOCX)

S2 Table Dogs lost to follow-up during the longitudinal study, stratified according to the reason for loss to follow-up, study area and evaluation time. (DOCX)

S3 Table Sand flies of the species *Lu*. *longipalpis* captured in the locality of Monte Gordo in the period between April and July 2015. (DOCX)
