# Supplementary material for: The mass use of deltamethrin collars to control and prevent canine visceral leishmaniasis: A field effectiveness study in a highly endemic area
Source: PLoS Negl Trop Dis. 2018 May 14;12(5):e0006496. doi: 10.1371/journal.pntd.0006496 (PMC5993122; doi:10.1371/journal.pntd.0006496)
Supplement: S1 Table — (DOCX) [file pntd.0006496.s001.docx]

|  | Dogs examined |
| --- | --- |
| Positive result for CVL | 23 |
| Negative result for CVL | 17 |
| Prevalence of CVL | **57.5%** |

* The animal was considered with LVC when it presented positive result in at least one of the tests performed (DPP, ELISA, qPCR and culture)
