# Supplementary material for: The mass use of deltamethrin collars to control and prevent canine visceral leishmaniasis: A field effectiveness study in a highly endemic area
Source: PLoS Negl Trop Dis. 2018 May 14;12(5):e0006496. doi: 10.1371/journal.pntd.0006496 (PMC5993122; doi:10.1371/journal.pntd.0006496)
Supplement: S2 Table — (DOCX) [file pntd.0006496.s002.docx]

| Reasons for loss to follow-up | Intervention area | | Control area | |
| --- | --- | --- | --- | --- |
|  | Between the 1st and 2nd survey | Between the 2nd and 3rd survey | Between the 1st and 2nd survey | Between the 2nd and 3rd survey |
| Positive animals with indication of euthanasia | 105 (38.7%) | 25 (20.2%) | 38 (45.2%) | 10 (16.7%) |
| Animals that presented allergic reactions to the collar with deltamethrin | 1 (0.4%) | 2 (1.6%) | --- | --- |
| Animals donated / sold / abandoned on the street | 10 (3.7%) | 15 (12.1%) | 2 (2.4%) | 4 (6.7%) |
| Animals escaped / disappeared / stolen | 9 (3.3%) | 4 (3.2%) | 0 | 2 (3.3%) |
| Animals died / were collected by the CCZ | 31 (11.4%) | 29 (23.4%) | 7 (8.3%) | 6 (10.0%) |
| Dog owner changed residence | 3 (1.1%) | 5 (4.0%) | 0 | 3 (5.0%) |
| Could not contact owners ^(1)^ | 106 (39.1%) | 43 (34.7%) | 35 (41.7%) | 0 |
| Refusal of owners | 6 (2.2%) | 1 (0.8%) | 2 (2.4%) | 35 (58.3%) |
| Total of lost-to-follow-up animals ^(2)^ | 271 | 124 | 84 | 60 |

^(1) 47 dogs that had been lost between the first and second survey in the intervention area and 16 dogs lost in the control area were evaluated again in the third survey^

^(2) There was no statistically significant difference in the total number of animals lost between the study areas (^*^p^* ^= 0.5 at both times)^
