# Supplementary material for: The mass use of deltamethrin collars to control and prevent canine visceral leishmaniasis: A field effectiveness study in a highly endemic area
Source: PLoS Negl Trop Dis. 2018 May 14;12(5):e0006496. doi: 10.1371/journal.pntd.0006496 (PMC5993122; doi:10.1371/journal.pntd.0006496)
Supplement: S3 Table — (DOCX) [file pntd.0006496.s003.docx]

| Months of capture | *Lu. longipalpis* captured | |
| --- | --- | --- |
|  | Intervention area | Control area |
| April | 0 | 0 |
| May | 7 | 2 |
| June | 23 | 9 |
| July | 0 | 0 |
| Total | **30** | **11** |
